# Entangled Speech: Speaking through oral microbes

**DOI:** 10.1080/13528165.2020.1882208

**Published:** 2021-03-09

**Authors:** Klaus Spiess, Maximilian Hauptmann, Lucie Strecker

**Keywords:** language, oral microbes, entanglement, diffraction

## Abstract

The backdrop for our performance is the entanglement of humans and their microbes. In detail we explore the relation between human-centered language and microbes, aiming to give their relationship more meaning and structure. The performance itself unfolds as an entanglement between the researcher, the research objects and the method of investigation. Oral microbes transgress the boundaries between themselves and human speakers. They react to individual phonemes with specific changes in their ecology, their needs authoring human voicescapes, disembodying and decoupling the voice from the rational and essentialist humanist subject. Both voice and microbes are agentially cut together-apart: what looks like a separation through the technical apparatus we use to enfold the intra-actions actually proves the mutual entanglement of both entities. With this cut, language loses its subject, its owner and sovereign, with phonemes and microbes becoming two co-hosts that contribute to the voicescape equally. The material apparatuses in our performances, which enhance and visualize non/human reactions, produce material phenomena through specific causal intra-actions. In its intra-activity, the matter is not a passive object to be observed and analyzed but the microbes becoming co-agents, taking part in the discursive practice. They are already material-discursive and that is, according to Barad, what it means to matter. Diffraction becomes a matter of differential entanglements, which do not intertwine or other the voice and microbes as separate entities, but prove their inseparability by becoming materially connected. Microbial entangled speech may be more important than has been assumed and has so far been overlooked as a connecting layer between the human body and its non-human inhabitants.

Recently we have been researching performance practices that question the ‘representationalism’ of language, which is considered to have rendered material realities inaccessible behind the linguistic systems that construct or represent them (Barad 2007). Representationalism conceives the world and its materiality in linguistic and conceptual terms, linked to a subjective and anthropocentric thinking that has been dominant in ontology and epistemology since Descartes and Kant; the human, by speaking and thinking, is still thought to construct the world. Because the dynamic and agential potential of materiality has been ignored, much research has lately been presented that foregrounds the material aspects of language (Freitas de 2014; Cavanaugh and Shankar 2017). In our performances we also raise the question of whether the human is indeed the exclusive ontological unit to start an epistemological enquiry into language. We explore the methods that would be necessary to shift to a non-anthropocentric ontology that places non-human beings, such as microbes inhabiting the human, at the centre of a performance. In the last decade especially, microbes have been considered to influence human evolution and behaviour at many levels (Rees, Bosch and Douglas 2018). In introducing oral microbes as co-hosts of human speech we challenge a traditional definition of language, considered as the one property of the human subject that necessarily excludes all other species from participation.

With Barad’s (2007) method of diffraction, we aim at microbes leaving the place of merely analysed and observed objects to become agents, self-involved entities, intra-acting with voice, language and speech, freeing them from the mastery of a human subject.

From this point of view, appealing questions for a performative set-up arise: How can we performatively apply the in-between of both the threat of partializing language as autonomous, detachable phonemes and the pleasure of voicing the ephemeral qualities of phonemes? To what extent can human speech and arbitrarily motivated words be disconnected from a human source or principles to introduce other than human sources? How can we explore the range of vocal and oral modalities as a way to foreground a politics of speech that includes both non-human participation and human identity? How can we create a precise microperformative set-up that explores entanglements of non-humans with spoken language?

In our earlier performances we have already experimented with dis/embodied speech from different perspectives. In *Spitparty* (Spiess and Strecker 2014) saliva took the role as semiotic material passable directly from mouth to mouth. In *Hare’s Blood* (Spiess and Strecker 2017) *Voicescapes* (Johnson 2004) stemming from capitalistic auction chants directed the survival of a living microbial artwork, and in a recent project we analysed immunologists’ gestures while they were explaining how microbes invaded the human body (Spiess *et al.* 2018).

For a most recent series of performance series titled *Microbial Keywording* (Spiess and Strecker 2019a, 2019b) the immediate entanglement of oral microbes with speech became a tested starting point.

## MICROBES CO – AUTHORING SPEECH

For *Microbial Keywording* we chose a certain microbial species, inhabiting the human mouth, which we here refer to more generally as ‘oral microbes’. The microbes chosen can create the best conditions for their ecology, in anticipating and memorizing changes of chemical-abiotic factors (oxygen, acids, temperature) (Mitchell *et al.* 2009), to which they adapt ecologically with cell death, sexual transformation and reproduction. Speakers’ breath and saliva therefore become the precondemn both for the microbes to thrive and the phonemes to be spoken. Oxygen, moistness and temperature in the mouth were the specific conditions that we related to the rhythmic alteration of salivation and breath during actual speech of the audience. The unique oral wetland of speakers became the base both for the microbes’ ecology and the phonemes’ vivacity.

To enhance the subtle alterations of saliva flow and acidity (Gilchrist and Furchtgott 1951) our audiences had to speak certain phonemes repetitively while their saliva was harvested. Acidity, oxygen flow and temperature were continually measured by sensors; the ecological changes of microbes were projected on a large wall screen using a dark field microscope, giving the whole performance room a more fluid emanation of voices entangled with microbes.

In order to enhance the audience’s oral microbes’ ability to memorize the microtraumatic phonemes, for a certain time an intricately designed technical apparatus exposed speakers’ microbes to microbial pheromones (Caudron and Barral 2013), which were added according to the spectrograms of the speakers’ phonemes. The highly specific ecological adaption of the microbes’ rates of reproduction and cell death remained although the pheromones were removed, favouring certain input phonemes and thereby altering their alphabetic order. The data shown on the screen were not inert, but continually changed depending on how the participants changed their input phonemes.

Eventually, after having ecologically adapted to the input phonemes, the microbes were returned to the speakers as a probiotic, a vial labelled with the respective ecologically adaptive phoneme, called a ‘phonetic mouthwash’.

## PLEASURES OF PRELINGUISTIC ARTICULATION

The participants were invited to choose phonemes from words with a certain personal meaning to them. Words could stem from their pet names, utterances of pain and love or communication with animals or toddlers. By whispering, speakers sometimes brought breath and words closer together, which LaBelle (2014: 147) called ‘to rest one on top of the other’. A more rhythmic intonation often allowed the speakers to mouth the words and (de-)form their articulators specifically. When enough interest had been triggered in the other audience members, the phonetic chanting shifted from very intimate personal utterances to collective ones.

**Figure:**
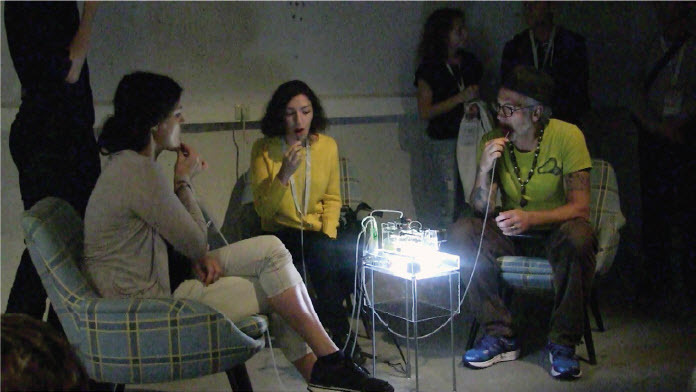
*Microbial Keywording*, Ars Electronica Linz, 2019. Saliva Harvest. © *Spiess*

When participants collectively babbled, gargled or sputtered speech became bumpy and language misconception was rapidly at work. The rhythms that evolved to explore the relationship between phonemes and saliva/air flow were a type of chanting that, through parameters such as pitch, vocal style and intensity, was often performed in a way that created a vocal soundscape, although sufficiently distant from a singing voice.

Intimate utterings became a ‘transitional phenomenon’ according to Winnicott’s (2005) theory: by their salivating, participants sometimes seemed to ‘suck’ from the phonemes they had chosen. Winnicott considered this kind of protolanguage calming, due to his impression that the infant felt that they created their own phonetic objects, representative of the absent mother, or in our performance the absent signified.

De Certeau (1996: 33) underscores this tension such utterances pose on language in general – as pleasures of prelinguistic mouth movements that unsettle the contours of meaning to suggest a project for what he addresses as a postlinguistic future based on a vocal utopia.

Since language acquisition is accompanied by a decline of the ability to recognize the differences between all phonemes in human language, these utterings made the audience more aware of phonemes as being representations of the microbes depicted on the screen.

**Figure:**
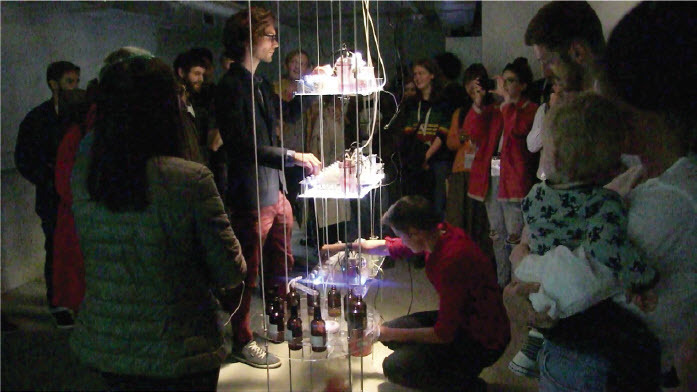
*Microbial Keywording*, Ars Electronica Linz, 2019. Chanting Audience with Arrangement of Single-use-bioreactors, Nutritional Fluids and Biosensors for Pheromone Addition. © *Spiess*

## FOCUSING THE JANUS FACE OF PHONEMES

The mechanism for generating the human voice involves many parts: the lungs with airflow, the vocal folds within the larynx and the articulators, which are the palate, tongue, cheeks, teeth, lips and salivary glands, which articulate and filter the sound emanating from the larynx. To enhance the saliva flow and microbial changes dependent on these articulations the audience was invited to embody phonemes by gesturing. This rehearsal also increased the participants’ awareness of articulating certain phonemes and consonants. At the beginning, the participants started using a repertoire of mouth actions that are characterized by the mouth ‘echoing’ or mirroring certain articulatory actions of the hands. For example, abrupt separating of the hands was accompanied by the oral syllable [pa] (Woll 2014). These articulations were neither derived from spoken words nor visually motivated but required a certain exhalation or inhalation of breath, usually with a change in mouth configurations during the articulation of the phoneme. The intention was to give the participants a basic feeling of situations of non-arbitrariness, where the hands ‘drive’ the mouth, and not the other way around, as in gestures accompanying speech. Our intention was to transfer speakers’ manual actions to oral ones and to convert ambivalent units of an iconic manual communication system into an apparent largely non-arbitrary vocal communication system to become a play for the participants.

## PERFORMING WITH ORAL MICROBES

Microbes in a speaker’s mouth clearly have many little-known capacities that the humans they inhabit do not. The Nobel laureate Lederberg (2006) claimed that the microbes seem to be much more interactive than humans are. Compared to human cultures, they reproduce more in collectives (Wloch-Salamon *et al.* 2017). Microbes intensely intermediate between the human and the surrounding ecosystems (Buzzini *et al.* 2017). The metabolism and sexual reproduction of microbes are sensitive to many alterations in the environment, including industrial profit-making, which all lead to rapid changes in microbial community life. When in danger of starving, microbes not only switch their metabolism from sugar to oxygen consumption but also their sexuality from energy-saving asexual self-cloning to sexual replication, to ensure their genetic diversity. It is not merely the case that the microbes’ sexual system is embodied. The oral microbes very being may be referred to as sexual: microbes are not only a sexually metamorphosing system in the human mouth; they optimize their sexuality to maximize their perception of the oral environment. Microbes signal themselves or a part of their community to die if they are starving and recycle their own cellular waste as food for other cells, guaranteeing the survival of the community and of the whole organism. With their volatiles, microbes have evolved sophisticated forms of attracting a transporting species and so have become true cosmopolitans. For microbes, as for language, circulation is survival.

With this wide scope of microbially acting entities the thriving power in constructing and defining the world are set far beyond the human subject.

It is this microbial ecology that is affected and in return affects in *Microbial Keywording*. Mouth microbes perceive phonemes with their very ecological being, which favours certain phonemes, deleting others. For oral microbes, substance and form, materiality and intelligibility, all being and all knowing, entail one another. Microbes in our mouths are not bound on a fixity of metabolic, sexual or migrating boundaries; it rather seems they intrinsically live the mutability of matter, and a sensing relation between them and the environment is *materiality enacted* (Barad 2007: 132–188).

*Microbial Keywording* pinpoints the highly specific emanations of pitch and tone to supply different life conditions to them. The subject passes on its suggested exclusive ownership of voice and speech to the microbes, to non-human capabilities living and acting in the human body, the human voice and speech thereby becoming an essential materialistic process, a materialist practice.

We refer to this entanglement of devices, microbes, saliva chemistry, voices and words from which certain ecological patterns emerge by reading through one another as an ‘eco-material or ‘ecolinguistic’ realm.

## VOICE: MICROBIAL MEMORY MATERIALLY ENACTED

In *Microbial Keywording* we reimagine language/speech with an ontology not of an individual rational ‘I’, but of a materialist discursive practice, with microbes as an important part of it. Speech, which emanates from an essentialist human speaker, is transformed by the needs of non-humans inhabiting the speaker’s mouth. Human speech becomes fragmented and shifts to an ecological non-human-human becoming. Such speech describes a disarticulated, dismantled organism that is an assemblage of non-human forces, needs, thresholds, conjunctions and intensities (Deleuze and Guattari 1987 [1980]: 177), a voice without a subject, which does not rely on the human being as the primary ontological unit of enquiry. Speech becomes a process of coupling and connecting different bodies, places, spaces, times, expressions and beings. This process creates a non-essentialist microbial layer of oral ecological cravings and longings.

We think of voice as a surface for the recording of the whole process/performance (Deleuze and Guattari 1983 [1972]:11), which includes the adaptations of the microbes, rather than thinking of voice as a singular process that seeks to record any subjective experience and to translate this experience into the form of a singular and individual voice. In contrast, the microbes transcend all boundaries between organisms before recording. Instead of archiving the voice in space and time like a dead object, these microbiological beings record a dehierarchized, microbially growing, sexual and metabolically constantly transforming part of voice.

All hope of breaking with the habits of the humanist subject is lost if the memory is static, the memory of the psyche, as the knowing subject experiences it. The microbial memory in *Microbial Keywording* is a different memory of voice. It becomes an ecological memory, a materialist memory, which within its cell membrane directly reacts to its entire environment. The voice is therefore a becoming of a different ontology, a microbial ontology, with a memory that carries a collective environmental past in its entirety and not only the cognitive past of the human it has inhabited. This memory includes life, which, according to Deleuze (2005 [1995]), can never be specified or linked to a self, which does not represent the ‘experience’ of individual subjects.

## MICROBES WITH A VOICE

In *Microbial Keywording* phonetically entangled microbes also became part of a conventional biological recording process in the Petri dish. This capability of infinite replication shifted the traditional structures of vocal authenticity. Whereas the experience of listening to stories ‘passed on from mouth to mouth’ (Benjamin 1968: 84) is highly individual, the biological recording in *Microbial Keywording* quickly became an experience of disembodiment, because the record by the Petri dish relied on microbes being passed while speaking, breaking the bodily connection between the speaker and their individual voice. When biological reproduction and cell death determined the outlasting of the voice, it became associated with a living grave marker.

*Microbial Keywording* made it possible to think about the dynamic and action, the life that is happening in our mouths within its own biological system. What was seen as a genuine creating subject before was now merely a co-host, a part of an intra-action that produced language. Moreover, thinking about language also meant thinking about ownership of this language. Who is speaking? Who does the voice that the audience heard belong to? Where does it come from? *Microbial Keywording* made clear that this question is not so easy to answer. It comes close to a ‘Voice without a subject’ that Mazzei (2016) derives from Deleuze and Guattari’s ‘Body without Organs’.

The term BwO originally was derived from Artaud’s work, who claimed that taking the organs away from the body in order to forget all its automatic reactions will set the body free at last (Artaud 1963). Artaud himself aimed for a theatre of gestures, sounds and screams, transcending and destroying language, meaning an obliteration of known signifiers. For Deleuze (1990), Artaud and his theatre of cruelty were the typical example of the schizophrenic, who is not interested in the surface of social norms or alphabetical language, who goes deeper and beyond, into the materiality of being. But Artaud’s concept of performance still centres on the human, looking for catharsis. To take the term Body without Organs seriously means getting a body as an assemblage of forces and non-human agents like microbes, which thrive, intra-act and take control of the body.

What are the forces and agents that constitute voice in *Microbial Keywording*? Microbes, pheromones and saliva flow. Indeed, with this practice the voice is set free, as Artaud demanded. Freed from the subject. Leaving established humanist patterns behind is what Artaud called cruel. From a theoretical perspective our performance possibly comes closer to Artaud’s idea of a Theatre of Cruelty, which he himself had conceptualized. Not humans, but microbes are the main characters in *Microbial Keywording*, the humanist view of body and voice being replaced by microbial-material intra-acting.

The Body without Organs is still a Body with Microbes, a body filled with sexually active, transforming, migrating, ever-changing microorganisms, which inhabit all organs and that have a decisive influence on their activities. But this new term, Body with Microbes, derived from Deleuze and Guattari, still places the human body in the position of the acting subject. Rather, microbes become a new kind of organ themselves, insofar as their actions are vital for the human body and all its other organs. It is not the microbes that are an *órganon* for the human body, but the human body is an *órganon* for the microbes, a body where they can display their various capabilities. Not a Body without Organs nor a Body with Microbes, but Microbes with (a) Body; not Voice without a Subject, but Microbes with a Voice.

## A DIFFRACTIVE READING OF ORAL MICROBES

Within the *Microbial Keywording* performance, microbes intra-act as phonemes and vice versa. According to their very being, microbes are a speech apparatus, and phonemes are not some conceptualized linguistic representations made up by the mind but living beings. They live, interfere, influence and change all the time; for example, the microbes change their sexuality in response to the available phonetic triggers.

As mentioned above, the technical apparatus used in *Microbial Keywording* works as the necessary tool of diffraction. Phenomena emerge in the intra-action between the technical apparatus, not used as an instrument of objective observation but as a constitutive part of the performance and the microbes. The phenomena are the smallest units of an analysis, not observed by the technical instruments, but created within them, emergent in the field between the apparatus and the microbes. One cannot observe a phenomenon with the analysing instruments, because the phenomenon does not precede the experiment, but arises from it (Barad 2007). Nothing just *is*, passively waiting to be observed, but everything emerges from intra-actions between instrument and object, between phoneme improvisation, voice-spectrograms, Petri dishes, bioreactors, microbial adaptation and phonetic probiotic. The diffractive method of investigating is not just cutting apart – the microbes from the human mouth – but *cutting together-apart* in one movement, thus emphasizing the potential of intra-action between the microbes in a Petri dish and the phonemes we speak. First, the microbes are separated from their natural habitat – the human mouth – but it is this cut that makes clear how entangled microbes and phonemes are, with the human mouth as the field of this entanglement. By diffractive reading, the entanglement of speech, microbes and the whole technical apparatus in *Microbial Keywording* is not some necessary experimental setup to gain knowledge about the real essence of microbes or the definite behaviour of speech, but becomes a performance itself, from which new relations between phonemes and microbes unfold and emerge.

**Figure:**
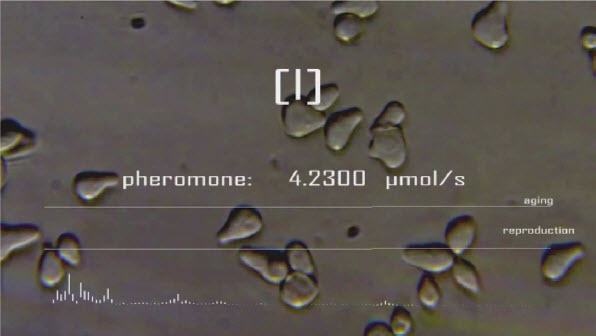
*Microbial Keywording*, Ars Electronica Linz, 2020. Spectrogram lead Pheromone Addition and Replicating Microbes, Wall Screen. © *Spiess*

As Barad’s (2014) brittlestar does not cut itself off from the environment, even if it loses one of its limbs, Barad queries whether this separated part belongs to the brittlestar or the environment. Connectivity does not necessarily require physical proximity (Barad 2014), the microbes do not belong to their surroundings, to people or to voices – all these entities are intra-actively connected, materially staged and carry out discursive practices such as speech. The microbes are an ‘externality within’ that closes the gap and allows one to perceive human language in its materiality, which changes our relationship with it.

**Figure:**
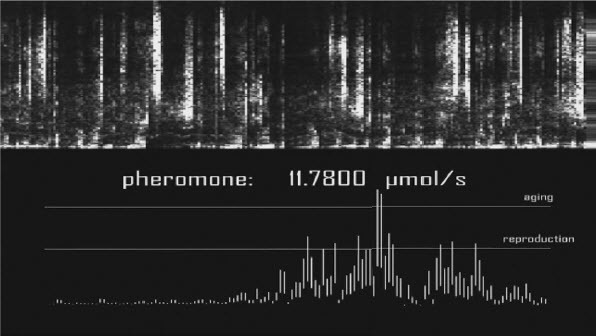
*Microbial Keywording*, Ars Electronica Linz, 2019. Spectrogram lead Pheromone Addition and Voice Sonogram, Wall Screen. © *Spiess*

## CONCLUDING THOUGHTS

In our current society, with problems like climate change, destruction of nature and the loss of biodiversity, Timothy Morton (2010: 274) emphasizes how important it is to see nature as an intimate process that happens not outside but inside and with us: ‘When the environment becomes intimate … it is decisively no longer an environment, since it is no longer just happens around us: that’s the difference between weather and climate.’ To acknowledge this quality of nature opens space for the microbes as intimate in *Microbial Keywording* – what is more intimate than microbes changing their sexuality, dying and reproducing themselves in our mouths?

We may not be able to escape linguistic representationalism completely, but through *Microbial Keywording* the audience is enabled to re-think the concept of language and speaking as process of another intimacy. What can we learn about our speech-acts from the intra-action between microbes and phonemes that is the centre of our performance? In the tradition of philosophers like Austin and Butler, language is performative, and speaking is acting. But at their core these ideas are still anthropocentric, leaving language and speaking to human subjects alone. Newer ideas, like Barad’s intra-action, seek to shift the focus from constructing our world through language to recognizing the active role of objects like atoms or observational instruments, making research not an objective measurement but a performance itself. *Microbial Keywording* goes even further, as it explicates the materiality of language and speaking, raising the question about its own ontological status.

We can no longer think about language, speech and voice without shifting our focus to its materials and objects. Then we can get to voice as a genuine ontological entity that is living, changing and independent of any subject. As is so often the case, literature very early anticipated a language without a subject, for example, in Francois Rabelais’s (2008) frozen words scene or, as Ivan Kreilkamp writes, in Conrad’s *Heart of Darkness*: ‘Those lines from *Heart of Darkness* – “The horror! The horror!” – announce the dawning of an awareness that language might function with no clear connection to its human source’ (Kreilkamp 1997: 211).

However, these depictions remain within the boundaries of language. As described above, our performance transcends these borders and gives the participant an experience of a language spoken not by a human subject, but performed by microbes, that is by the constitutive elements of speech. It is, as one might say, hearing language speak itself, microbes choosing their phonemes, forming the human into a tool (an *órganon*) to create an environment that is most suitable for them.
